# Granite dust application to hemp – variety-specific impacts on growth and cannabinoid production

**DOI:** 10.1038/s41598-023-49529-9

**Published:** 2023-12-14

**Authors:** N. K. Hillier, L. Voscort, L. Zamlynny, W. Hillier, N. Faraone

**Affiliations:** 1 Department of Biology, Acadia University, 33 Westwood Ave, Wolfville, NS Canada; 2 Department of Chemistry, Acadia University, 6 University Ave, Wolfville, NS Canada

**Keywords:** Plant sciences, Analytical chemistry

## Abstract

The hemp industry has grown exponentially with the recent legalization of *Cannabis sativa* in Canada. With this new market expansion, there is an increased need for hemp plants, particularly for production of cannabinoids. Growing concerns regarding pesticide residues in commodities for human consumption, as well as global demand for fertilizer has increased consumer demand for natural products as alternatives to synthetic agrochemicals and pest management strategies. The objective of this study was to investigate the potential for using different composite granite dusts applied as soil amendments in improving *C. sativa* growth, and cannabinoid production (specifically, cannabidiol and cannabidiolic acid). We selected three varieties of industrial hemp with low yield production of cannabidiol (Fibranova, CFX-2, and Katani) and one variety with high yield production of cannabidiol (Cherry Blossom). Varieties were planted in potting soil amended with zero, five or ten percent granite dust mixture, and assayed for growth characteristics, and cannabinoid composition. Among tested cannabis varieties, results suggest that improvements to flower growth (> 44% mass) and cannabinoid production (> 2.5 fold or > 145%) from application of granite dust were evident in one variety of fibre hemp, CFX-2. Overall, this work suggests there may be selective benefits to soil applications of granite dust composites to improve hemp propagation, and that degree of improvement to cannabinoid production vary between varieties of hemp.

## Introduction

Hemp is an important crop globally, in food, fibre and pharmaceutical production^1^. Scientifically known as *Cannabis sativa* ssp. *sativa* L., hemp stands as a versatile and multifaceted crop with the potential to offer to a wide array of traditional and innovative industrial applications^2^. Its appeal lies in its straightforward and resource-efficient cultivation methods^3^, coupled with the sustainability of its products^4^. These factors collectively propel the anticipated growth and diversification of the hemp crop in the future.

In Canada, the use of cannabis products was legalized in October 2018. Since then, there has been a major growth of a market for cannabis production for both recreational and medicinal applications. Hemp plants can be used for a wide variety of applications, such as food, paper, plastic concrete, source of pharmaceutical active ingredients, etc.^5,6^. Furthermore, hemp's ecological advantages extend beyond its growth phase. It is known for its ability to sequester carbon dioxide (CO_2_) from the atmosphere, making it a promising player in carbon offset strategies and climate change mitigation^7^.

Important areas for development in this market are finding safe and effective ways to grow cannabis plants organically and managing the increased risk of pests affecting quality and quantity of plants in mass production facilities. Maintenance of a stable supply chain of cannabis products for recreational and medical markets will require new, innovative, and sustainable tools for pest management. Furthermore, rigorous regulation by Health Canada regarding the use of unauthorized pest control products for *C. sativa* creates an urgent need to validate and certify novel pest management products.

Cannabidiol (CBD) is a phytocannabinoid with no psychoactive properties, accounting for the majority of the chemical composition of floral and flower extracts in high-yield cannabis and industrial hemp varieties. Preliminary clinical research on CBD has demonstrated benefits in treating a range of anxiety, cognition, movement disorders, as well as pain^8^. Cannabidiolic acid (CBDA), the precursor of CBD, is another abundant chemical compound native to cannabis and hemp plants; this phytocannabinoid is converted into CBD through a thermal decarboxylation process^9^.

Maintaining and expanding the industrial hemp industry in Canada and abroad requires optimization of production, including cultivation techniques, choice of varieties for regions and climates, and harvest and processing strategies which increase yield^1–4^. As a growth industry in Canada, maintenance of a stable supply chain of CBD for recreational and medical markets will require development of novel tools to enhance CBD production. Techniques which optimize hemp and cannabis growth and enrich the quantity and quality of CBD could significantly improve yield and value for farmers^1^. Appropriate use of fertilizers and soil amendments is critical for enhancing growth of many crops, as well as for enhancing production of key target materials (i.e., fruit, seed, grains, fibre, or flowers)^10,11^. Soil amendments may function to increase fertility, improve water and nutrient uptake, and increase overall crop performance by altering the physicochemical properties of soils. In return, this may promote the increased production of the main active ingredients in cannabis; the important cannabinoids THC (tetrahydrocannabidiol) and CBD^12,13^. We have shown that soil amendment with granite dust can improve fruit yield and provide insect resistance in zucchini^14^, and as well, soil amendment provided protection from two-spotted spider mite damage in tomatoes^15^. Similarly, recent work has demonstrated that solid amendment with rock phosphate dust can significantly increase biomass and THC production in *C. sativa*^16^. Granite dust is rich in silicon in the form of SiO_2_^15^ along with other oxides. Several beneficial properties have been linked to the exposure of plants to Si-rich dusts, such improving stressed plant behaviour exposed to heavy metals^17^ and resistance to drought, salinity, and heat^18^. As such, the application of granite dust may also provide conditions for naturally boosting the production of flowers and associated cannabinoids in *C. sativa*.

This study investigates the potential for application of granite dust composites for improving growth of *C. sativa* ssp. *sativa* (industrial hemp). Specifically, it was hypothesized that soil amendment would improve plant growth and cannabinoid production in four different varieties of hemp.

## Materials and methods

### Plants and varieties

All experiments were performed under a Health Canada research licence (CTLS: LIC-1P82KSVKZ2-2019; Acadia University) issued to N. Kirk Hillier. Four varieties of hemp, *C. sativa* ssp. *sativa*, were selected for the experiment: Fibranova (fibre variety, low yield CBD), CFX-2 (fibre variety, low yield CBD), Katani (fibre variety, low yield CBD), and Cherry Blossom (high yield CBD, feminized). Seeds were acquired from Hemp Genetics International Inc. (HGI), (Saskatoon, SK, Canada—CFX-2 and Katani), Schiavi Seeds (Lexington, KY, USA—Fibranova), and CBD Seed Labs (Carlsbad, CA, USA—Cherry Blossom). Using these varieties, experiments were conducted to determine the effects of granite dust application.

### Treatment

Granite dust was provided by Heritage Memorials Ltd. (Windsor, NS, Canada) as a by-product of granite-cutting process. It is composed by a mixture of granite dusts and is a source of silicon in the form of amorphous silica, SiO_2_ (constituting approximately 60% of the overall composition). Full chemical composition of granite dust was reported in Faraone et al.^15^. Two mixtures of granite dusts (silicate and aluminosilicate) with different % of SiO_2_ were tested (the rose dust has a much greater SiO_2_ relative to the composite—specific elemental analysis is proprietary of the company and under a non-disclosure agreement) with an average particle size 60–20 mm.

### Growth experiment 1: low CBD-yield varieties

Measurement of growth and overall production in both male and female plants was recorded for the following treatments using three low-CBD yield hemp varieties: Fibranova; CFX-2; Katani. Seeds of each variety were planted directly in potting soil (VPW-420, ASB Greenworld, Pointe Sapin, NB, Canada – this potting media is based upon on natural sphagnum peat moss combined with dolomitic lime stone, perlite, vermiculite, mycorrhizae, a natural wetting agent plus a starter charge of nutrients) into 3-gallon pots without additional nutrition or fertilizers, and subjected to one of the following treatments (N = 16 per each treatment, variety, and sex (male/female) of plant. Note that up to 25 plants were within each treatment, however, we reduced the number to 16 in order to have matching numbers of males and females in treatment groups for this experiment):

- Control.
- 5% w/w composite granite dust as a soil amendment.
- 10% w/w composite granite dust as a soil amendment.

Plants were maintained under greenhouse conditions (Temperature 25C ± 3 °C; RH 50 ± 10%), with supplemental full spectrum growth lights (15 h day length set from 5am to 8pm on high intensity discharge lamps) and watered every second day at the K.C. Irving Environmental Sciences Centre at Acadia University (Wolfville, NS, Canada) for the duration of the study. PAR (photosynthetically active radiation) was maintained between 400 and 600 during vegetative growth, and increased to 600–800 during bloom). For each plant measurements of stem length, number of leaves, number of flowers (females), terminal flower length, as well as the dry mass of leaves, flowers, and roots were taken at within one week of 90% bloom (after 85 days of growth from August to November 2021). Hemp stems, leaves, flowers, and roots were collected from all plants, coarsely chopped, placed in paper bags (one bag/tissue/plant) and placed in a drying oven at 50 ± 2 °C until completely dried (7–10 days). Means for all variables were recorded for male and female plants.

### Growth experiment 2: high CBD-yield varieties

Growth and production in high CBD-yield Cherry Blossom variety plants were monitored using the same methods as described in Experiment 1. Plants were subjected to the same preceding treatments, as well as a 5% rose dust soil amendment (N = 15 of each treatment). This variety is feminized so no sex comparisons were made. For each plant, measurements of stem length, number of leaves, number of flowers (females), as well as the dry mass of leaves, flowers, and roots were made at maturity. Hemp stems, leaves, flowers, and roots were collected and processed, following the same protocol as previously described.

### Chemical extraction and high performance liquid chromatography (HPLC) analysis of CBD and CBDA extracts

CBD (purity 98.66%) and CBDA (purity 97.88%) certified standards were purchased from Cerilliant Corporation Inc. (Round Rock, TX, USA). Acetonitrile and methanol HPLC grade were purchased from Fisher Scientific Inc. (Fair Lawn, NJ, USA). Absolute ethanol was purchased from Commercial Alcohols Inc. (Brampton, ON, Canada). Water was filtrated with a Milli-Q® Type 1 Ultrapure Water System (Millipore Sigma, Oakville, ON, Canada).

Dried hemp flowers were ground into a fine powder using a mill grinder and 0.25 g portions were weighed for extractions. Five (5) mL of absolute ethanol were added to the weighed hemp powder and stirred for 20 min. Following this, the supernatant was collected, and an additional 5 mL of ethanol was added to the plant material. This process was repeated three times until 15 mL of supernatant were collected. Solvent was removed by rotary evaporation and the weight of extracted material was recorded. The extract was then dissolved in 4 mL of absolute methanol, filtered using a 0.45 μm filter (Chromatographic Specialities Inc., Brockville, ON, Canada), and stored at 4 °C until further analysis.

The HPLC profiles were acquired on an Agilent 1260 Infinity II HPLC equipped with DAD G1315D, quaternary pump G1311A, column thermostat G1316A and thermostatted autosampler G1329A (Agilent Technologies Inc., Santa Clara, CA, USA). The profiles were recorded at 230 nm. The analytical column was an Infinity Lab Poroshell 120 EC-C18 (4.6 × 100 mm, 2.7 μm), with an Infinity Lab Poroshell UHPLC guard column 120 EC-C18 (3.0 mm), both from Agilent Technologies. The separations were conducted in gradient condition using as mobile phase a mixture of water-acetonitrile. The [water: acetonitrile] solvent ratio was [40:60] from 0:00 to 7:00 min, then moved to [23:77] from 7:01 to 8:20 min, and finally [5:95] from 8:21 to 9:00 min. The column was re-equilibrated under initial conditions for 4 min. The flowrate was 1 mL/min and total runtime was 9 min. Standard curves for CBD and CBDA were prepared by diluting 1 mg/mL certified standards. Serial dilutions were prepared in methanol ranging from 0.1 to 100 μg/μL. Each hemp extract was diluted 20 times (50 μL of sample in 950 μL of methanol) and analyzed by HPLC UV–Vis. Samples and standards were injected in triplicates. The identity of isolated cannabinoids was confirmed by comparing retention times (HPLC) and spectroscopical data (UV–Vis) with reference compounds and literature^19^.

### Data analysis

Statistical analyses were conducted with RStudio Version 2022.12.0 + 353 (RStudio Inc. 2022). Growth characteristics were compared and cannabinoid content among different varieties and treatments was analyzed with generalized linear model followed by analysis of variance (ANOVA) and pairwise Tukey’s test (P < 0.05) using the lme4^20^, lsmeans^21^, and multcomp^22^ packages. For growth studies, independent analyses were conducted for sex, variety, treatment, and variety x treatment interactions.

### Ethical approval

All experiments and collection of plant material, complies with institutional, national, and international guidelines and legislation, and with the IUCN Policy Statement on Research Involving Species at Risk of Extinction and the Convention on the Trade in Endangered Species of Wild Fauna and Flora.

## Results and discussion

### Results: plant growth experiments

Soil amendment with granite dust at 5% w/w and 10% w/w dosages and applied to different varieties of *C. sativa* yielded selective improvement to growth characteristics versus control plants of the same varieties (Tables 1 and 2). Significant differences were noted between varieties in the number of leaves, flowers, flower length, and the dry mass of stems, leaves, flowers, and roots.

**Table 1:** Results of general linear model comparing various growth characteristics of low-CBD yield varieties of *Cannabis sativa* subjected to two dosages of composite granite dust as a soil amendment.

Sex	P values from GLM analyses for various growth characters			
	Plant characters	Variety	Treatment	Variety x treatment
Female	Stem length (cm)	p = 0.59	p = 0.20	p = 0.47
	Number of leaves	p < 0.05	p = 0.06	p = 0.06
	Number of flowers	p < 0.001	p < 0.05	p < 0.01
	Terminal flower length (cm)	p < 0.001	p = 0.42	p < 0.05
	Stem dry mass (g)	p < 0.001	p = 0.45	p = 0.49
	Leaf dry mass (g)	p < 0.001	p = 0.07	p = 0.08
	Flower dry mass (g)	p < 0.001	p = 0.18	p < 0.05
	Root dry mass (g)	p = 0.13	p = 0.06	p = 0.52
Male	Stem length (cm)	p = 0.31	p < 0.05	p = 0.42
	Number of leaves	p = 0.34	p = 0.49	p = 0.57
	Stem dry mass (g)	p = 0.08	p = 0.50	p = 0.69
	Leaf dry mass (g)	p = 0.13	p = 0.52	p = 0.98
	Root dry mass (g)	p < 0.05	p = 0.39	p = 0.71

Analyses were conducted separately for sex, and variety, treatment, and variety x treatment interaction were used as variables in analyses (N = 16).

**Table 2:** Growth characteristics of three low-CBD yield varieties of *Cannabis sativa* subjected to two dosages of composite granite dust as a soil amendment.

Variety	Treatment	Stem length (cm) ± SE	Number of leaves ± SE	Number of flowers ± SE	Terminal flower length (cm) ± SE	Stem dry mass (g) ± SE	Leaf dry mass (g) ± SE	Flower dry mass (g) ± SE	Root dry mass (g) ± SE
Male									
Katani	Control	85 ± 5.6	9 ± 1.2	–	–	8 ± 0.4	9 ± 0.4	–	8 ± 0.1
	5%	102 ± 11.2	9 ± 1.4	–	–	9 ± 0.7	10 ± 0.8	–	8 ± 0.2
	10%	95 ± 8.3	9 ± 1.1	–	–	9 ± 0.4	9 ± 0.5	–	8 ± 0.1
CFX-2	Control	104 ± 5.1	12 ± 1.6	–	–	9 ± 0.4	9 ± 0.3	–	8 ± 0.1
	5%	114 ± 15.3	9 ± 2.7	–	–	10 ± 1.5	10 ± 1.1	–	8 ± 0.5
	10%	93 ± 7.0	11 ± 1.8	–	–	9 ± 0.4	10 ± 0.5	–	9 ± 1.2
Fibranova	Control	89 ± 8.6	7 ± 0.7	–	–	10 ± 0.5	9 ± 0.15	–	9 ± 0.4
	5%	134 ± 25.3	8 ± 0.3	–	–	10 ± 0.3	9 ± 0.6	–	9 ± 0.0
	10%	98 ± 9.9	12 ± 1.5	–	–	11 ± 0.7	11 ± 0.2	–	10 ± 0.5
Female									
Katani	Control	105 ± 7.4	12 ± 1.7	78 ± 11.9	11 ± 1.1	20 ± 2.0	17 ± 1.1	37 ± 1.8	13 ± 1.0
	5%	105 ± 6.8	13 ± 1.6	67 ± 9.9	12 ± 1.6	19 ± 2.5	15 ± 1.5	31 ± 2.5	12 ± 0.7
	10%	110 ± 11.4	18 ± 2.8	82 ± 7.3	8 ± 1.0	20 ± 2.9	19 ± 2.0	37 ± 3.1	14 ± 0.9
CFX-2	Control	97 ± 15.9	10 ± 1.6	47 ± 14.8	9 ± 0.9	18 ± 4.5	15 ± 2.1	27 ± 3.9	13 ± 1.2
	5%	116 ± 11.0	16 ± 2.2	101 ± 13.5*	8 ± 0.9	24 ± 3.6	21 ± 2.4	36 ± 4.8*	14 ± 1.3
	10%	121 ± 7.0	10 ± 2.4	95 ± 9.1*	10 ± 1.2	24 ± 0.5	18 ± 1.6	39 ± 2.0*	16 ± 1.3
Fibranova	Control	108 ± 6.4	9 ± 1.7	15 ± 1.0	17 ± 1.9	14 ± 0.9	11 ± 0.6	25 ± 1.4	13 ± 0.6
	5%	123 ± 5.5	12 ± 0.5	15 ± 0.7	12 ± 0.6	13 ± 0.8	12 ± 0.7	22 ± 1.4	13 ± 0.8
	10%	109 ± 5.8	11 ± 0.7	14 ± 0.7	16 ± 0.9	15 ± 1.1	13 ± 0.7	24 ± 1.6	14 ± 0.7

Asterisks indicates growth characteristics (flower number and dry mass) which were significantly increased following soil amendment with 5 and 10% w/w composite granite dust; *p* < *0.05*; N = 16).

In female plants, Katani variety plants treated with 10% w/w granite dust had significantly more leaves than controls. While not significant, number of leaves and leaf dry mass exhibited a trend to increased amounts in all treatments of female plants (Tables 1 and 2). Fibranova produced significantly fewer flowers than Katani and CFX-2 varieties, which were not different from one another. Furthermore, CFX-2 variety plants treated with 5 or 10% w/w dosages of granite dust produced significantly more flowers than control plants (p < 0.05). Terminal flowers were significantly longer in Fibranova variety plants at maturity versus CFX-2 and Katani varieties (p < 0.05), however granite dust treatments did not provide significant increases to terminal flower length within varieties versus control plants (p = 0.34). Stem and leaf dry masses were significantly lower for Fibranova plants versus other varieties (p < 0.05; p < 0.05) but were otherwise not affected by granite dust treatment. Flower dry mass was similarly lower for Fibranova, but 5–10% w/w soil amendment resulted in significant increases in flower mass in CFX-2 plants (p < 0.05). No significant differences were found in dry root mass between varieties or treatments (Table 1). There was an overall trend towards 5% w/w soil amendment generally having decreased growth in all categories, but this was largely not significant.

For male plants, when treated with 5% w/w granite dust, Fibranova produced a larger dry root mass than Katani (p < 0.05). No other effects were significant in male plants.

For the high-yield CBD variety, Cherry Blossom, an additional treatment of 5% w/w rose granite dust was also tested (Table 3). Overall, 5% w/w composite granite dust provided the greatest mean number and mass of flowers, longest stem length, and largest number and dry weight of leaves. However, differences between all varieties and treatments for growth characteristics measured were not statistically significant (p < *0.05*).

**Table 3:** Growth characteristics of Cherry Blossom hemp varieties.

Treatment (% w/w)	Mean stem length (cm) ± SE	Mean # of leaves ± SE	Mean # flowers ± SE	Mean dry weight flowers (g) ± SE	Mean dry weight leaves (g) ± SE
F (3,38)	0.62	1.31	1.30	2.39	1.60
p (0.05)	0.60	0.28	0.29	0.08	0.20
Control (0%)	131 ± 8.5	47 ± 1.9	429 ± 83.6	9 ± 0.3	57 ± 6.1
5%	138 ± 5.4	49 ± 2.1	681 ± 143.5	11 ± 1.1	67 ± 5.9
10%	131 ± 6.6	46 ± 2.8	409 ± 67.9	9 ± 0.6	52 ± 3.1
Rose 5%	126 ± 4.1	43 ± 2.1	534 ± 111.3	9 ± 0.9	66 ± 6.4

No significant differences were noted between control, soil amendment with 5% and 10% composite granite dust, or 5% rose dust (general linear model using a one-way ANOVA and means separated with Tukey’s test; *p* < *0.05*; N = 15).

### Results: extraction and analyses of CBD and CBDA

Analysis of relative levels of CBD and CBDA in treated/untreated plants was performed by HPLC–UV/Vis using previously built calibration curves (Table S1). Using the same treatments as in Experiment 1, flowers from selected varieties were extracted and analyzed for CBD and CBDA content. The results obtained from HPLC analysis are summarized in Tables 4 and 5. Katani and Fibranova varieties did not contain significant amounts of CBDA and are therefore not included, and Cherry Blossom plants subjected to rose granite dust treatment were not tested for CBD or CBDA content.

**Table 4:** CBD and CBDA (μg/mg ± SE) content across two hemp varieties treated with different concentrations of granite dust soil amendments.

Variety	Treat	CBD (μg/mg) ± SE	*t*	*p*	CBDA (μg/mg) ± SE	*t*	*p*
CFX-2	Control	3.27 ± 0.280	–	–	0.112 ± 0.0134	–	–
	5%	6.48 ± 1.52	2.24	0.0306*	0.227 ± 0.0236	2.95	0.00524*
	10%	8.00 ± 0.827	3.30	0.00197*	0.269 ± 0.0392	4.03	0.000228*
Cherry Blossom	Control	7.78 ± 0.945	–	–	24.5 ± 2.22	–	–
	5%	5.91 ± 0.395	− 1.848	0.262	30.9 ± 3.72	1.370	0.523
	10%	8.04 ± 0.879	0.253	0.994	21.6 ± 2.38	− 0.607	0.929
	5% rose	7.26 ± 1.91	− 0.515	0.955	26.6 ± 4.25	0.440	0.971

Significant effects are noted with an asterisk (*p* < *0.05*; N = 15; Tukey’s test).

**Table 5:** CBD (μg/mg ± SE) content across two hemp varieties treated with different concentrations of granite dust soil amendments (*p* < *0.05*; N = 15; Tukey’s test).

Variety	Treat	CBD (μg/mg) ± SE	t	p
Katani	Control	4.42 ± 0.67	–	–
	5%	5.98 ± 0.99	− 1.503	0.3174
	10%	2.87 ± 0.41	1.495	0.3208
Fibranova	Control	2.99 ± 0.58	–	–
	5%	2.24 ± 1.49	0.740	0.744
	10%	2.01 ± 0.24	0.568	0.839

## Discussion

Many agricultural crops are treated with silicon-based fertilizers used for protection against biotic and abiotic factors and optimal growth of plants^23^. Granite dust is an easily accessible commercial fertilizer because in its dry and wet compositions it is active and has similar properties to DE, and other silicon-based products^15,24^. As such, it may provide an alternative treatment for improving crop production in fiber and pharmaceutical production of *C. sativa*. Crushed rocks have been successfully used for enhancing soil fertility, resulting in elevated agricultural productivity, the restoration of depleted areas, water purification, and carbon sequestration^7,25^. The favorable impacts reported from the treatment of rock dust has been linked to the presence of silicon which is known to have numerous beneficial effects on plants exposed to abiotic and biotic stresses^26^. Furthermore, the exposure of plants to silicon enhanced the production of secondary metabolites^27–29^. Based on these previous findings, we hypothesized that soil amendment with granite dust would improve growth and cannabinoid production in multiple hemp varieties.

Soil amendment improved flower growth in CFX-2 variety *C. sativa* plants. No significant changes were noted in other treatments and varieties tested, however there were distinct variety-dependent differences in most growth characteristics measured, with female Fibranova plants typically producing fewer, longer flowers, and having significantly lower stem, leaf, and flower mass versus other low-CBD yield varieties. All varieties have distinct CBD and CBDA profiles, with high-CBD industrial hemp varieties such as Cherry Blossom generally having higher quantities of CBD and CBDA than fiber or dual-purpose varieties. Furthermore, it is important to note that cumulative amount of CBD is represented by the combined CBD and CBDA amount extracted, as CBDA is precursor.

In terms of quantitative changes in cannabinoid production, the application of granite dust as soil amendment significantly increased the production of CBD and CBDA only for CFX-2. The amount of CBD doubled after a 5% w/w application of granite dust, and almost tripled after a 10% w/w application of granite dust. CBDA production was also positively impacted by the application of granite dust as soil amendment, reporting a similar trend with nearly doubled CBDA production after the granite dust application. The other varieties did not show any significant changes in cannabinoid production after being exposed to granite dust, and particularly for Fibranova and Katani, the concentration of CBDA was below the detection limit, therefore it was not possible to quantify any variation for this target analyte. The impact of the type of fertilizer and concentration used on cannabinoid profile has been reported in different hemp varieties. Cherry blossom, as the highest cannabinoid producer, was reported to be significantly impacted by the increment of synthetic fertilizer (Anderson et al., 2021). Future work may investigate the interactions of granite dust application with supplementary fertilizer treatments or with the application of silicon, which may synergize to further improve growth or cannabinoid production^27^.

Growth and cannabinoid production of Cherry Blossom high CBD-variety plants were not significantly improved by soil amendment with granite dust. It should be further noted that Cherry Blossom plants did not generally perform as well as CFX-2 plants under the planting conditions (this was evident in control and treated plants), with slower development and overall reduced biomass produced by this variety. As above, supplementary fertilization may have been a limiting factor in Cherry Blossom production, which may have obscured any positive effects of granite dust soil amendment.

Differences in growth and outcomes between these varieties are not surprising as these have been selected for key growing characteristics. Katani and CFX-2 variety of hemp were chosen for analyses as they are both industrial fiber hemp varieties and both produce relatively high amounts of CBD, the non-psychoactive component of *C. sativa*^30,31^. CFX-2 plants are typically shorter than most hemp plants but contain a high yield of seeds; Katani plants are similar and produce many larger seeds^32^. Differential response to granite dust soil amendment may suggest that these varieties have different capacity for utilizing the physicochemical changes induced by granite dust application. As such this study provides an important inference to understand that variety differences need to be considered when evaluating soil amendment efficacy and type and concentration of fertilizer used^33^.

All plants respond to and take up nutrients and other substances differently. Plant species differ in how they take up silicon and how they store it in their tissues^15,34^. By stimulating pro-cambial activity in hemp plants this can cause increased production of biomass when silicon is present^35^. The hormones in plants are important in the production of pro-cambial cells^35^, and different amounts of phytohormones in Katani and CFX-2 plants could be the reason for the 16 shorter plants of CFX-2 planted with 5% w/w granite dust. If silicon is a key factor for enhancing plant growth, it is worth noting that dicots, such as *C. sativa*, do not accumulate much silicon in the tissues compared with monocots^15,26,35^.

In this study, plants were treated with granite dust and tested for growth under optimal conditions in a greenhouse. However, silicon that is found in granite dust has been purported as a factor to combat plant stress and drought effects by acting as a barrier and increasing the rigidity of the tissues and cells in the plants^26,35^. Furthermore, if cell wall stability is stronger, then leaves are less likely to be penetrated by pests’ mouthparts for feeding, pathogens, and other environmental stressors^15,35^.

## Conclusion

This study shows promising results for the use of granite dust to improve growth of hemp plants and CBD/CBDA production when applied as a soil amendment. The relative improvement is variety-specific, with notable increases in flower production and CBD/CBDA content seen in concert specifically with CFX-2 variety hemp plants. Future work should investigate the mechanisms for variety specific responses and optimization of granite dust application technology to enable widespread use of this material in cannabis propagation.

### Supplementary Information

Supplementary Table S1.

## Data Availability

All data from this study are available from the corresponding author on reasonable request.

## References

[1] Desanlis F, Cerruti N, Warner P, Arnaud PBSAL (2013). Hemp agronomics and cultivation. Hemp: Industrial Production and Uses.

[2] Amaducci S, Scordia D, Liu FH, Zhang Q, Guo H, Testa G, Cosentino SL (2015). Key cultivation techniques for hemp in Europe and China. Ind. Crops Prod..

[3] Tang K, Struik PC, Yin X, Thouminot C, Bjelková M, Stramkale V, Amaducci S (2016). Comparing hemp (*Cannabis*
*sativa* L.) varieties for dual-purpose production under contrasting environments. Ind. Crops Prod..

[4] Calzolari D, Magagnini G, Lucini L, Grassi G, Appendino GB, Amaducci S (2017). High added-value compounds from Cannabis threshing residues. Ind. Crops Prod..

[5] Oomah BD, Busson M, Godfrey DV, Drover JCG (2002). Characteristics of hemp (*Cannabis*
*sativa* L.) seed oil. J. Food Chem..

[6] Tang K, Struik PC, Yin X, Calzolari D, Musio S, Thouminot C, Bjelková M, Stramkale V, Magagnini G, Amaducci S (2017). A Comprehensice study of planting density and nitrogen fertilization effect on dual-purpose hemp (*Cannavis sativa* L.) cultivation. Ind. Crops Prod..

[7] Parvez AM, Lewis JD, Afzal MT (2021). Potential of industrial hemp (*Cannabis*
*sativa* L.) for bioenergy production in Canada: Status, challenges and outlook. Renew. Sust. Energ. Rev..

[8] Scuderi C, Filippis DD, Iuvone T, Blasio A, Steardo A, Esposito G (2009). Cannabidiol in medicine: A review of its therapeutic potential in CNS disorders. Phytother. Res..

[9] Citti C, Pacchetti B, Vandelli MA, Forni F, Cannazza G (2018). Analysis of cannabinoids in commercial hemp seed oil and decarboxylation kinetics studies of cannabidiolic acid (CBDA). J. Pharm. Biomed. Anal..

[10] Moran-Salazar RG, Sanchez-Lizarraga AL, Rodriguez-Campos J, Davila-Vazquez G, Marino-Marmolejo EN, Dendooven L, Contreras-Ramos SM (2016). Utilization of vinasses as soil amendment: Consequences and perspectives. SpringerPlus.

[11] Wylie SE, Ristvey AG, Fiorellino NM (2021). Fertility management for industrial hemp production: Current knowledge and future research needs. Glob. Change Biol. Bioenergy.

[12] Ahmed B, Hijri M (2021). Potential impacts of soil microbiota manipulation on secondary metabolites production in cannabis. J. Cannabis Res..

[13] Santunionea, G., Turia, E., Parisb, R. & Grassic, G. Production and use of co-composted biochar as soil amendment for *Cannabis sativa* sp. Growth. In European Biomass Conference and Exhibition Proceedings (pp. 113–117). ETA-Florence Renewable Energies (2020). 10.5071/28thEUBCE2020-1DV.1.3

[14] Faraone N, Hillier NK (2020). Preliminary evaluation of a granite rock dust product for pest herbivore management in field conditions. Insects.

[15] Faraone N, Evans R, LeBlanc J, Hillier NK (2020). Soil and foliar application of rock dust as natural control agent for two-spotted spider mites on tomato plants. Sci. Rep..

[16] De Prato L, Ansari O, Hardy GESJ, Howieson J, O’Hara G, Ruthrof KX (2023). Physiological and cannabinoid responses of hemp (*Cannabis sativa*) to rock phosphate dust under tropical conditions. Funct. Plant Biol..

[17] Luyckx M, Hausman JF, Guerriero G, Lutts S (2023). Silicon reduces zinc absorption and triggers oxidative tolerance processes without impacting growth in young plants of hemp (*Cannabis*
*sativa* L.). Environ. Sci. Pollut. Res..

[18] Beerling DJ, Leake JR, Long SP, Scholes JD, Ton J, Nelson PN, Bird M, Kantzas E, Taylor LL, Sarkar B, Kelland M, DeLucia E, Kantola I, Müller C, Rau GH, Hansen J (2018). Farming with crops and rocks to address global climate, food and soil security. Nat. Plants.

[19] Madej K, Kózka G, Winiarski M, Piekoszewski W (2020). A simple, fast, and green oil sample preparation method for determination of cannabidioloic acid and cannabidiol by HPLC-DAD. Separations.

[20] Bates, D., Maechler, M., Bolker, B., Walker, S., Christensen, R. H. B., Singmann, H., Dai, B., Scheipl, F., Grothendieck, G. & Green, P. Package ‘lme4’. URL http://lme4.r-forge.r-project.org (2009).

[21] Lenth R, Lenth MR (2018). Package ‘lsmeans’. Am. Stat..

[22] Hothorn, T., Bretz, F., Westfall, P., Heiberger, R. M., Schuetzenmeister, A., Scheibe, S. & Hothorn, M. T. Package ‘multcomp’. Simultaneous inference in general parametric models. Project for Statistical Computing, Vienna, Austria (2016).

[23] Reynolds OL, Padula MP, Zeng R, Gurr GM (2016). Silicon: potential to promote direct and indirect effects on plant defense against arthropod pests in agriculture. Front. Plant Sci..

[24] Faraone N, MacPherson S, Hillier NK (2018). Evaluation of repellent and insecticidal properties of a novel granite dust production in crop production. J. Pest Sci..

[25] Ramos CG, Hower JC, Blanco E, Oliveira MLS, Theodoro SH (2022). Possibilities of using silicate rock powder: An overview. Geosci. Front..

[26] Luyckx M, Hausman JF, Lutts S, Guerriero G (2017). Silicon and plants: Current knowledge and technological perspectives. Front. Plant Sci..

[27] Ahanger MA, Bhat JA, Siddiqui MH, Rinklebe J, Ahmad P (2020). Integration of silicon and secondary metabolites in plants: A significant association in stress tolerance. J. Exp. Bot..

[28] Sivanesan I, Park SW (2014). The role of silicon in plant tissue culture. Front. Plant Sci..

[29] Zhong JJ, Seki T, Kinoshita SI, Yoshida T (1992). Effects of surfactants on cell growth and pigment production in suspension cultures of Perilla frutescens. World J. Microbiol. Biotechnol..

[30] Darby, H., Gupta, A., Cummings, E., Ruhl, L. & Ziegler, S. Industrial Grain Hemp Variety Trial [Extension Report]. *Northwest Crops & Soils Program* 7 (2017).

[31] Hammami N, Privé JP, Joly DL, Moreau G (2021). Associations between cannabinoids and growth stages of twelve industrial hemp varieties grown outdoors in Atlantic Canada. Ind. Crops Prod..

[32] Miyan, S. Industrial Hemp Variety Trials. AgriFutures Australia publication no. 22–145 (2022).

[33] Anderson SL, Pearson B, Kjelgren R, Brym Z (2021). Response of essential oil hemp (*Cannabis*
*sativa* L.) growth, biomass, and cannabinoid profiles to varying fertigation rates. PLoS One.

[34] Ma JF (2004). Role of silicon in enhancing the resistance of plants to biotic and abiotic stresses. J. Soil Sci. Plant Nutr..

[35] Luyckx M, Hausman JF, Lutts S, Guerriero G (2017). Impact of silicon in plant biomass production: Focus on bast fibres, hypotheses, and perspectives. Plants.

