# Supplementary material for: Granite dust application to hemp – variety-specific impacts on growth and cannabinoid production
Source: Sci Rep. 2023 Dec 14;13:22254. doi: 10.1038/s41598-023-49529-9 (PMC10721882; doi:10.1038/s41598-023-49529-9)
Supplement: Supplementary file 1 — Supplementary Table S1. [file 41598_2023_49529_MOESM1_ESM.docx]

**Supplemental Material:**

**Table S1:** Calibration curve equations for CBD and CBDA and their respective *R^2^* values.

| **Component** | **Concentration range** | **Linear equation** | ***R^2^* value** |
| --- | --- | --- | --- |
| CBD | 0.1-100 μg/μL | y = 51.554x – 3.7776 | *0.9996* |
| CBDA | 0.1-100 μg/μL | y = 116.44x + 5.3981 | *0.9999* |
